# IGF-1/IGF-1R/FAK/YAP Transduction Signaling Prompts Growth Effects in Triple-Negative Breast Cancer (TNBC) Cells

**DOI:** 10.3390/cells9041010

**Published:** 2020-04-18

**Authors:** Damiano Cosimo Rigiracciolo, Nijiro Nohata, Rosamaria Lappano, Francesca Cirillo, Marianna Talia, Domenica Scordamaglia, J. Silvio Gutkind, Marcello Maggiolini

**Affiliations:** 1 Department of Pharmacy, Health and Nutritional Sciences, University of Calabria, 87036 Rende, Italy; damianocosimo.rigiracciolo@unical.it (D.C.R.); rosamaria.lappano@unical.it (R.L.); francesca.cirillo@unical.it (F.C.); marianna.talia@unical.it (M.T.); scordamagliadomenica1@gmail.com (D.S.); 2 MSD K.K., Tokyo 102-8667, Japan; nijiro.nohata@merck.com; 3 Department of Physics, University of Calabria, 87036 Rende, Italy; 4 Department of Pharmacology, Moores Cancer Center, University of California, San Diego, La Jolla, CA 92093, USA

**Keywords:** TNBC, IGF-1, IGF-1R, FAK, YAP, OSI-906, VS-4718, verteporfin

## Abstract

Triple-negative breast cancer (TNBC) is an aggressive breast tumor subtype that currently lacks targeted treatment options. The role played by the insulin-like growth factor-1 (IGF-1) and its cognate receptor IGF-1R in TNBC has been reported. Nevertheless, the molecular mechanisms by which the IGF-1/IGF-1R system may contribute to TNBC progression still remains to be fully understood. By computational analysis of the vast cancer genomics information in public databases (TCGA and METABRIC), we obtained evidence that high IGF-1 or IGF-1R levels correlate with a worse clinical outcome in TNBC patients. Further bioinformatics analysis revealed that both the focal adhesion and the Hippo pathways are enriched in TNBC harboring an elevated expression of IGF-1 or IGF-1R. Mechanistically, we found that in TNBC cells, the IGF-1/IGF-1R system promotes the activation of the FAK signal transduction pathway, which in turn regulates the nuclear accumulation of YAP (yes-associated protein/yes-related protein) and the expression of its target genes. At the biological level, we found that the IGF-1/IGF-1R-FAK-YAP network cascade triggers the growth potential of TNBC cells, as evaluated in different experimental systems. Overall, our results suggest that the IGF-1/IGF-1R/FAK/YAP axis may contribute to the progression of the aggressive TNBC subtype.

## 1. Introduction

Breast cancer is the most frequently diagnosed malignant disease among women worldwide and is the main cause of cancer-related deaths [1]. Meaningful progress has been recently made in the early detection and treatment of breast cancer. However, a wide number of patients may relapse as a consequence of distant metastasis, in particular those affected by the highly malignant triple-negative breast cancer (TNBC) subtype [2]. The TNBC, which represents approximately 15–20% of all diagnosed breast carcinomas, is characterized by aggressive biological features leading to a poor clinical outcome [3,4]. Considering the worst prognosis and the lack of targeted treatments of TNBC, major efforts are required for setting novel therapeutic strategies [4].

The insulin-like growth factor-1 (IGF-1) binding to the insulin-like growth factor receptor-type 1 (IGF-1R) activates different transduction events, promoting the growth and survival of multiple types of cancer cells [5,6,7]. In particular, the IGF-1/IGF-1R system mediates stimulatory effects in malignant cells through various pathways such as the phosphatidyl-inositol-3 kinase (PI3K)/AKT1, the mammalian target of rapamycin (mTOR), and the mitogen-activated protein kinase (MAPK) [8,9,10]. Indeed, alterations in IGF-1/IGF-1R signaling as well as aberrant IGF-1/IGF-1R expression have been associated with cell proliferation, anti-apoptotic, epithelial to mesenchymal transition (EMT) and migratory effects in different tumors [11,12,13]. For instance, high IGF-1R expression and elevated IGF-1 circulating levels have been correlated with an increased breast cancer risk and poor prognosis in breast cancer patients [14,15]. In addition, IGF-1R expression has been associated with a shorter survival of TNBC patients with respect to estrogen receptor- (ER)-positive breast tumor patients [16]. In this regard, a high IGF-1 gene expression signature mediating cancer proliferation and survival has been assessed in TNBC cells and primary TNBC specimens, hence suggesting a rationale for targeting the IGF-1/IGF-1R system in this highly aggressive breast cancer subtype [17].

Focal adhesion kinase (FAK) is a non-receptor cytoplasmic protein-tyrosine kinase engaged by several upstream signaling systems including integrins, G protein-coupled receptors (GPCRs), and growth factor receptors, in which FAK regulates important processes contributing to both normal and cancer cell growth [18]. Mechanistically, the activity of FAK results in the phosphate transfer from ATP to tyrosine residues of substrate proteins like src, paxilin, p130Cas, cortactin, and β-catenin [19]. Furthermore, FAK may act as a scaffold protein forming phosphorylation-independent complexes together with other cellular regulatory proteins [20]. Upon activation, FAK triggers downstream events that are involved in different features of cancer progression such as proliferation, survival, invasion, and angiogenesis [21]. In this regard, it should be mentioned that FAK over-expression has been linked to a worse prognosis in TNBC patients [22]. Furthermore, FAK inhibition has been shown to counteract the progression of TNBC and its metastatic potential, strengthening, therefore, the therapeutic option to target FAK in TNBC lesions [23].

The Hippo pathway is a highly conserved transduction pathway that exerts key roles in the regulation of organ size, tissue regeneration, immune responses and tumor development [24]. The core components of the Hippo pathway is a kinase cascade consisting of both the tumor suppressor Hippo kinase MST1/2 that associates with the SAV1 adaptor protein and the LATS1/2 kinases that associate with MOB1 [25]. The formation of these protein complexes promotes the phosphorylation of the transcription co-activators YAP (yes-associated protein/yes-related protein) and TAZ [25], preventing thereafter both nuclear shuttle of YAP/TAZ and their capability to activate the transcription factor TEA domain (TEAD-family members) [25,26]. Alterations of the Hippo pathway cascade may increase the YAP/TAZ nuclear localization and the consequent induction of gene expression leading to the progression of different types of tumors, including breast cancer [27,28,29]. Of note, the over-expression of YAP has been found to contribute to both radio-resistance and early relapse in TNBC patients [29,30]. Therefore, small-molecules inhibitors have been developed in order to abolish the YAP- and TAZ-TEAD interaction and consequently to halt the progression of diverse types of tumors [27,28].

On the basis of the aforementioned data and recent findings showing the involvement of the YAP/TAZ pathway in the action of FAK in tumor cells [31], we investigated the transduction signaling triggered by the IGF-1/IGF-1R system through both FAK and YAP in TNBC cells. Here, we provide novel mechanistic insights regarding the stimulatory function of the IGF-1/IGF-1R/FAK/YAP network on the growth responses of TNBC cells.

## 2. Materials and Methods

### 2.1. Analysis of Publicly Available Genomics and Transcriptomic Databases of Breast Cancer

The clinical significance of IGF-1 and IGF-1R was assessed by RNA-seq data of Breast Cancer Cohort of TCGA (The Cancer Genome Atlas: https://cancergenome.nih.gov/) [32] and microarray data of METABRIC [33]. TNBC was defined by the lack of expression of ER, progesterone receptor (PR), and human epidermal growth receptor 2 (HER2) receptors by immunohistochemistry (IHC). The gene expression data from TCGA and METABRIC were retrieved in November 2019 from cBioPortal (http://www.cbioportal.org/) [34]. The Z-scores of mRNA expression data and clinical sample information corresponding to breast cancer patients were collected from cBioPortal. The IGF-1/IGF-1R high group (either mRNA Z-score more than zero) and the IGF-1/IGF-1R low group (both mRNA Z-scores equal or less than zero) were analyzed by Kaplan–Meier survival curves and log-rank statistics. To identify the pathways potentially associated with IGF-1/IGF-1R, the GeneCodis3 pathway analysis was performed (http://genecodis.cnb.csic.es/) [35] for over-expressed genes in the IGF-1/IGF-1R high TNBC group. Then, to assess the networks regulated by IGF-1/IGF-1R signaling, genes were analyzed by KEGG (Kyoto Encyclopedia of Genes and Genomes) Pathway Database [36]. Gene set enrichment analysis (GSEA) was performed to identify enrichment pathways using open source software v4.0 (http://software.broadinstitute.org/gsea/index.jsp).

### 2.2. Chemicals and Drugs

Insulin-like growth factor-1 (IGF-1) and PI3K inhibitor Wortmannin (WM) were purchased from Sigma-Aldrich (Milan, Italy). The dual insulin-like growth factor 1 receptor (IGF-1R)/insulin receptor (IR) inhibitor OSI-906 (Linsitinib) was obtained from Tocris Bioscience (Space, Milan, Italy). Focal Adhesion Kinase selective inhibitor VS-4718 (PND-1186) was bought from Santa Cruz Biotechnology (DBA, Milan, Italy). The YAP/TEAD complex suppressor Verteporfin was purchased from Med Chem Express (DBA, Milan, Italy). All the aforementioned compounds were dissolved in dimethyl-sulfoxide (DMSO) except IGF-1 which was dissolved in water.

### 2.3. Cell Cultures

MDA-MB 231 cells were obtained from ATCC (Manassas, VA, USA), whereas SUM159 cells were kindly provided by Dr. W.T. Khaled (University of Cambridge, UK). Cells were used less than 6 months after thawing and routinely tested and authenticated according to the ATCC suggestions. MDA-MB 231, MDA-MB 231 PTK2 WT and MDA-MB 231 PTK2 KO cells (see below for technical details) were maintained in DMEM/F12 (Dulbecco’s modified Eagle’s medium) (Life Technologies, Milan, Italy) with phenol red, supplemented with 5% FBS and 100 μg/mL of penicillin/streptomycin. SUM159, SUM159 PTK2 WT and SUM159 PTK2 KO cells (see below for technical details) were maintained in DMEM/F12 (Dulbecco’s modified Eagle’s medium) (Life Technologies, Milan, Italy) with phenol red, supplemented with 1 μg/mL of insulin, 1 μg/mL of hydrocortisone, 5% FBS and 100 μg/mL of penicillin/streptomycin. Cells were grown in a 37 °C incubator with 5% CO_2_. Cells to be processed for immunoblot and RT-PCR assays were switched to medium without serum and phenol red 24h before treatments.

### 2.4. Focal Adhesion Kinase (FAK) CRISPR/Cas9 Genome Editing

Short guide RNA (sgRNA) sequence targeting human PTK2 gene encoding FAK was built using the CRISPR.mit.edu gRNA design (http://www.genome-enginnering.org) and then cloned into the pSpCas9 (BB)-2A-Puro (px459) vector (kindly provided by Gutkind’s Lab, Moores Cancer Center, University of San Diego (UCSD), California, USA according to the protocol described by Ran et al. [37]. The PTK2 sgRNA sequence designed was as follows: sgPTK2: 5′-TGAGTCTTAGTACTCGAATT-3′. MDA-MB-231 and SUM159 cells were transiently transfected with the plasmid sgRNA targeting PTK2 gene by Lipofectamine LTX (Life Technologies, Milan Italy). Upon a 36h transfection, cells were selected in a medium containing 1 µg/mL puromycin dihydrochloride (Sigma-Aldrich, Milan, Italy). After the puromycin selection, the puromycin-resistant colonies were picked and expanded in regular medium. Immunoblots and immunofluorescence staining for FAK and Y397-FAK proteins were performed in order to evaluate the FAK knockout efficiency.

### 2.5. Western Blotting Analysis

1 × 10^5^ MDA-MB 231, MDA-MB 231 PTK2 WT/KO, SUM159 and SUM159 PTK2 WT/KO cells were grown in 10 cm dishes. After 48 h, cells were exposed to the treatments and then lysed as previously described [38]. Equal amounts of whole protein extract were electrophoresed through a reducing SDS/8 and 10% (*w*/*n*) polyacrylamide gels, electroblotted onto nitrocellulose membranes (Amersham Biosciences, GE Healthcare, Milan, Italy), and probed with the following primary antibodies purchased from Cell Signaling Technology (Milan, Italy): Y1131-IGF-1Rβ (D6D5L), Y397-FAK (D20B1), FAK (3285S), S473-AKT (D9EXP), S127-YAP (4911S), and YAP (D8H1X). The following primary antibodies were purchased from Santa Cruz Biotechnology (DBA, Milan, Italy): IGF-1R (7G11), AKT 1/2/3 (H-136), Cyr61 (H-78), and β-actin (AC-15) and the following primary antibody purchased from Origene (DBA, Milan, Italy): CTGF (TA806803). Proteins were detected by horseradish peroxidase-linked secondary antibodies (Santa Cruz Biotechnology, DBA, Milan, Italy) and then revealed using the ECL™ Western Blotting Analysis System (GE Healthcare, Milan, Italy).

### 2.6. Immunoprecipitation Assay

1 × 10^5^ MDA-MB 231 and SUM159 cells were grown in 10 cm dishes. After 48 h, cells were exposed to treatments, washed and lysed using 500 μL RIPA buffer with protease inhibitors (1.7 mg/mL aprotinin, 1 mg/mL leupeptin, 200 mmol/L phenylmethylsulfonyl fluoride, 200 mmol/L sodium orthovanadate and 100 mmol/L sodium fluoride). Samples were then centrifuged at 13,000 rpm for 10 min and protein concentrations were determined using Coomassie (Bradford) protein assay. Proteins (250 μg) were then incubated for 2 h with 900 μL of immunoprecipitation buffer with inhibitors, 2 μg of anti-IGF-1R or anti-FAK antibodies and 20 μL of protein A/G agarose immunoprecipitation reagent (Santa Cruz Biotechnology, DBA, Milan, Italy). Samples were then centrifuged at 13,000 rpm for 5 min at 4 °C to pellet the beads. Pellets were washed four times with 500 μL of PBS and centrifuged at 13,000 rpm for 5 min at 4 °C. Supernatants were collected, resuspended in 20 μL RIPA buffer with protease inhibitors, 2X SDS sample buffer and heated to 95 °C for 5 min. Samples were then run on 10% SDS-PAGE, transferred to nitrocellulose and probed with primary antibodies. Western blot analysis and ECL detection were performed as described above.

### 2.7. RNA Extraction and Real-Time PCR

1 × 10^5^ MDA-MB 231 and SUM159 cells were grown in 10 cm dishes. After 48 h, cells were exposed to treatments and total RNA was extracted from cell cultures using the TRIzol commercial kit (Life Technologies, Milan, Italy) according to the manufacturer’s protocol. RNA was quantified spectrophotometrically and quality checked by electrophoresis through agarose gels stained with ethidium bromide. Only samples that were not degraded and showed clear 18 S and 28 S bands under UV light were used for RT-PCR. Total cDNA was synthesized from the RNA by reverse transcription using the murine leukemia virus reverse transcriptase (Life Technologies, Milan, Italy), following the protocol provided by the manufacturer. The expression of selected genes was quantified by real-time PCR using Step One™ sequence detection system (Applied Biosystems Inc., Milan, Italy), following the manufacturer’s instructions. Gene-specific primers were designed using Primer Express version 2.0 software (Applied Biosystems. Inc., Milan, Italy) and are as follows: human CTGF Fwd: 5′-ACCTGTGGGATGGGCATCT-3′ and Rev.: 5′-CAGGCGGCTCTGCTTCTCTA-3′; human Cyr61 Fwd: 5′-AAATCCCCCGAACCAGTC-3′ and Rev.: 5′-GGGCCGGTATTTCTTCACACT-3′; 18S Fwd: 5′-GGCGTCCCCCAACTTCTTA-3 and Rev.: 5′-GGGCATCACAGACCTGTTATT-3′. Assays were performed in triplicate and the RNA expression values were normalized using 18S expression and then calculated as fold induction.

### 2.8. Plasmids, Luciferase and Transfections Assays

The 8xGTIIC-luciferase reporter (Addgene #34615, originally from Stefano Piccolo’s Lab) was kindly provided by Dr. J. S. Gutkind [39]. The CTGF luciferase reporter plasmid p (−1999/+36)-Luc (CTGF-luc), based on the backbone of vector pGL3-basic (Promega) was a gift from Dr. B. Chaqour [40]. The Cyr61-luc luciferase reporter construct was provided by Dr. Xialong Yang, Department of Pathology and Molecular Medicine, Queen’s University, Kingston, Ontario [41]. The Renilla luciferase expression vector pRL-TK (Promega, Milan, Italy) was used as internal transfection control. MDA-MB 231 and SUM159 TNBC cells (1 × 10^5^) were plated into 24-well dishes with 500 μL/well culture medium containing 5% FBS. Cell medium was replaced on the day of transfection with serum-free medium and transfection was performed using X-tremeGENE 9 DNA Transfection Reagent as recommended by the manufacture (Sigma-Aldrich, Milan, Italy) and a mixture containing 0.5 μg of each reporter plasmid and 5 ng of pRL-TK. After 12h, cells were treated with IGF-1 alone or in combination with the IGF-1R inhibitor OSI-906, the FAK inhibitor VS-4718 or the YAP/TEAD complex suppressor Verteporfin and incubated for 12h. Luciferase activity was measured using the Dual Luciferase Kit (Promega, Milan, Italy) according to the manufacturer’s recommendations. Firefly luciferase activity was normalized to the internal transfection control provided by the Renilla luciferase activity. Normalized relative light unit values obtained from cells treated with vehicle (DMSO, <0.1%) were set as 1-fold induction upon which the activity induced by treatments was calculated. For gene silencing assay, the short hairpin (sh)RNA construct to knock down the expression of CTGF and the unrelated shRNA control construct have been described previously [42]. Briefly, MDA-MB 231 and SUM159 cells were transfected by using X-treme GENE 9 DNA Transfection Reagent (Roche Diagnostics, Milan, Italy) for 24 h before treatments with a control vector and a specific shRNA sequence for each target gene.

### 2.9. Immunofluorescence Microscopy

5 × 10^4^ MDA-MB 231 and SUM159 cells were grown on 6 well plates. When reached 50% confluence, cells were serum-deprived for 12 h and then treated for 30 min with IGF-1. Next, cells were fixed in 4% paraformaldehyde for 15 min at room temperature, permeabilized with 0.2% Triton X-100, washed three times with PBS and incubated overnight with or without (negative control) a rabbit primary antibody against Y397-FAK (Cell Signaling Technology, Milan, Italy). After incubation, the wells were extensively washed with PBS and incubated with donkey anti-rabbit IgG-FITC (1:400; purchased from Alexa Fluor, Life Technologies, Milan, Italy) for 1 h at room temperature. Finally, cells were washed with PBS and incubated in PBS buffer containing 4′, 6-diamidino-2-phenylindole dihydrochloride (DAPI) (1:1000; Sigma-Aldrich, Milan, Italy) 10 min at room temperature for nuclear staining. Images showing focal adhesion points among the cells were acquired on the Cytation 3 Cell Imaging Multimode Reader (BioTek, Winooski, VT, USA) and analyzed using the software Gen5 (BioTek). The same procedure was also applied for FAK (Cell Signaling Technology, Milan, Italy) staining in MDA-MB 231 PTK2 WT and KO as well as in SUM159 PTK2 WT and KO. A total of 10 images for each condition was detected on the Cytation 3 Cell Imaging Multimode Reader (BioTek) and analyzed using the software Gen5 (BioTek).

### 2.10. YAP Nuclear Staining

5 ×10^4^ MDA-MB 231 cells were grown on 6 well plates. When reached 50% confluence, cells were serum-deprived for 12 h and then treated for 30 min with IGF-1 alone or in combination with IGF-1R inhibitor OSI-906, PI3K inhibitor WM or FAK inhibitor VS-4718, as indicated. Next, cells were fixed in 4% paraformaldehyde for 15 min at room temperature, permeabilized with 0.2% Triton X-100, washed three times with PBS and incubated overnight with or without (negative control) a rabbit primary antibody against YAP (Cell Signaling Technology, Milan, Italy). After incubation, the wells were extensively washed with PBS and incubated with donkey anti-rabbit IgG-FITC (1:400; purchased from Alexa Fluor, Life Technologies, Milan, Italy) for 1 h at room temperature. Finally, cells were washed with PBS and incubated in PBS buffer containing 4′,6-diamidino-2-phenylindole dihydrochloride (DAPI), (1:1000), (Sigma-Aldrich, Milan, Italy) 10 min at room temperature for nuclear staining. Images showing YAP nuclear accumulation were acquired on the Cytation 3 Cell Imaging Multimode Reader (BioTek) and the percentage of cells with nuclear YAP was analyzed using the software Gen5 (BioTek). The same procedure was also applied for YAP (Cell Signaling Technology, Milan, Italy) nuclear staining in SUM159 PTK2 WT/KO cells. A total of 10 images for each condition was detected on the Cytation 3 Cell Imaging Multimode Reader (BioTek) and the percentage of YAP nuclear levels was analyzed using the software Gen5 (BioTek).

### 2.11. Cell Proliferation Assay

MDA-MB 231 and SUM159 TNBC cells (1 × 10^4^) were seeded in 24-well plates in regular growth medium, washed once they had attached, incubated in medium containing 2.5% charcoal-stripped FBS and then treated with IGF-1 alone or in combination with IGF-1R inhibitor OSI-906, FAK inhibitor VS-4718 or YAP/TEAD complex suppressor Verteporfin, as indicated. Medium and treatments were renewed every day. The proliferation rate was calculated counting the cells on day 5 using the Countess Automated Cell Counter, as recommended by the manufacturer’s protocol (Life Technologies, Milan, Italy).

### 2.12. Colony Formation Assay

MDA-MB 231 and SUM159 TNBC cells were cultured in regular growth medium to 90% confluence. Cells were then trypsinized, counted and 1 × 10^3^ cells were seeded in 6-well plates at 1 × confluence. The cells were treated with IGF-1 alone or in combination with the IGF-1R inhibitor OSI-906, the FAK inhibitor VS-4718 or the YAP/TEAD complex suppressor Verteporfin. MDA-MB 231 and SUM159 cells were transfected with shRNA control or shCTGF and then treated with IGF-1 (medium, transfections and treatments were renewed every 3 days). After 10 days, cells were washed with PBS, fixed in acetone:methanol (1:1) for 3 min at room temperature and then stained with crystal violet for 5 min. A total of 10 pictures for each condition was detected using a digital camera and colony number was measured by ImageJ program.

### 2.13. Spheroid Formation Assay

For spheroid generation, 100 μL/well of MDA-MB 231 and SUM159 cell suspensions (1 × 10^3^) were dispensed into 2% agar-coated 24-well plates. Three days after seeding, tumor spheroids (a single spheroid per well) were treated with IGF-1 alone or in combination with the IGF-1R inhibitor OSI-906, the FAK inhibitor VS-4718 or the YAP/TEAD complex suppressor Verteporfin. Medium and treatments replenishment were performed every 3 days. A total of 10 images for each condition was assessed on day 18 using a conventional inverted microscope, thereafter the spheroid area was determined by elaborating the images on IMAGE J software.

### 2.14. Statistical Analysis

The statistical analysis was performed using ANOVA followed by Newman-Keuls’ test to determine differences in means. *p* < 0.05 was considered as statistically significant.

## 3. Results

### 3.1. Focal Adhesion Is a Prominent Enriched KEGG Pathway Linked to the Expression of IGF-1/IGF-1R in TNBC

**Figure 1:**
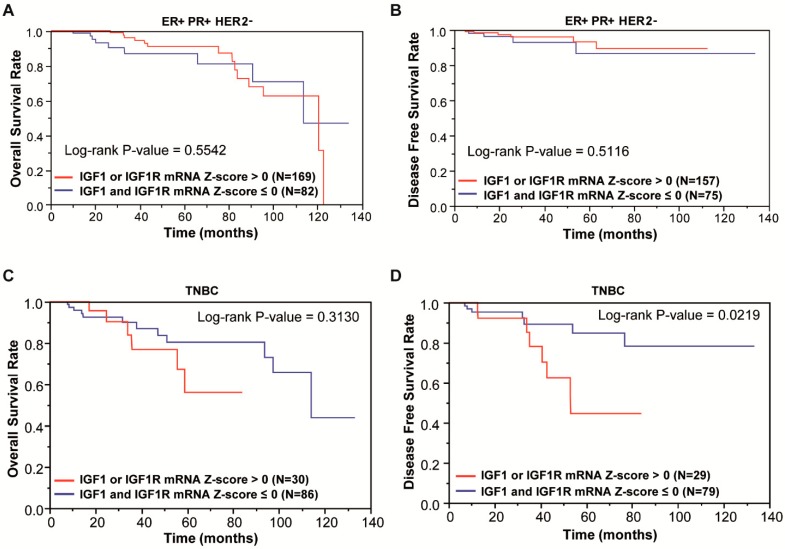
Clinical significance of IGF1/IGF1R (Insulin-like Growth Factor 1/IGF1 Receptor) system in breast cancer subtypes. (**A**) Overall Survival (OS) rate in ER/PR- (estrogen receptor/progesterone receptor)-positive and HER2- (human epidermal growth factor 2)-negative breast cancer patients according to the IGF1/IGF1R levels, as displayed by Kaplan-Meier plots with log-rank tests from the corresponding TCGA datasets. (**B**) Disease-Free Survival (DFS) rate in ER/PR-positive and HER2-negative breast cancer patients according to the IGF1/IGF1R levels, as displayed by Kaplan-Meier plots with log-rank tests from the TCGA dataset. (**C**) Overall Survival (OS) rate in triple-negative breast cancer (TNBC) patients according to the IGF1/IGF1R levels, as displayed by Kaplan-Meier plots with log-rank tests from the TCGA dataset. (**D**) Disease-Free Survival (DFS) rate in TNBC patients according to the IGF1/IGF1R levels, as displayed by Kaplan-Meier plots with log-rank tests from the TCGA dataset.

Alterations in the IGF-1/IGF-1R-mediated signaling have been associated with the development and progression of hormone-related tumors, including breast cancer [43,44]. In addition, the IGF-1/IGF-1R system has been implicated in the onset of mammary tumorigenesis [13] and the biological features of the highly malignant TNBC [45,46]. To date, IGF-1R has been detected approximately in 40% of TNBC [47] and correlated with a poor clinical outcome of this group of patients [16]. On the basis of the aforementioned findings, we began our study assessing the clinical significance of the IGF-1/IGF-1R expression in both the ER/PR-positive and HER2-negative breast cancer subtypes and the TNBC cohort. By mining the RNA-sequencing data of the Breast Cancer Cohort of TCGA (The Cancer Genome Atlas: https://cancergenome.nih-gov/) and microarray data of METABRIC, we evaluated the Kaplan-Meier survival rates of patients grouped according to high expression levels of IGF-1 or IGF-1R (or both) (mRNA Z-score more than 0) and low expression levels of IGF-1 and IGF-1R (mRNA Z-score equal or less than 0). In ER/PR-positive and HER2-negative breast cancer patients, the overall and disease-free survival rates did not evidence significant differences between high and low IGF-1/IGF-1R expression groups (Figure 1A,B). As it concerns the TNBC patients, the overall survival rate was found to be reduced, although not in a significant manner, and remained high with respect to the low IGF-1/IGF-1R group (Figure 1C). Of note, the DFS rate was found significantly decreased in TNBC patients with high IGF-1/IGF-1R expression with respect to those exhibiting low IGF-1/IGF-1R levels (Figure 1D).

**Figure 2:**
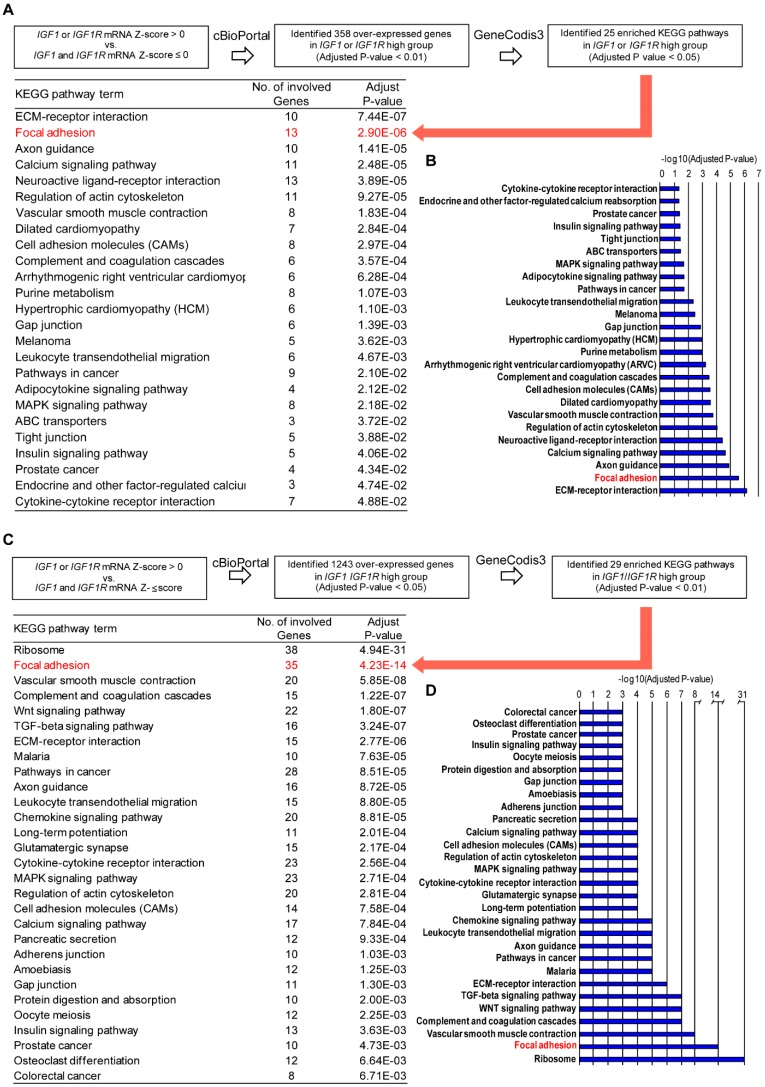
Focal adhesion gene signature is a prominent enriched pathway in TNBC patients with high IGF1/IGF1R expression levels. (**A**) In silico analysis of IGF1/IGF1R-regulated pathways in TNBC patients in the TCGA cohort. The over-expressed genes (n. 358) in the IGF1/IGF1R high group with adjusted *p*-value 0.01 (Student’s t-test with Benjamini-Hochberg procedure) compared to the IGF1/IGF1R low group were chosen from the results of TNBC in the TCGA cohort. The genes were then analyzed and categorized with the KEGG Pathway Database using the GeneCodis3 pathway analysis program with the threshold of adjusted *p*-value < 0.05. Red arrow indicates Focal Adhesion as a prominent KEGG pathway. (**B**) Bar graph of the significant enrichment KEGG pathways in IGF1/IGF1R high group. (**C**) In silico analysis of IGF1/IGF1R-regulated pathways in TNBC patients of the METABRIC dataset. The over-expressed genes (n. 1243) in IGF1/IGF1R high group with adjusted *p*-value < 0.05 (Student’s t-test with Benjamini–Hochberg procedure) compared to IGF1/IGF1R low group were chosen from the results of TNBC in the METABRIC dataset. The genes were then analyzed and categorized with the KEGG Pathway Database using the GeneCodis3 pathway analysis program with the threshold of adjusted *p*-value < 0.01. Red arrow indicates Focal Adhesion as a prominent KEGG pathway. (**D**) Bar graph of the significant enrichment KEGG pathways in IGF1/IGF1R high group.

Next, by Genecodis3 pathway analysis, we sought to provide novel evidence regarding the IGF-1/IGF-1R relevant molecular signatures in TNBC cohorts. In this vein, we first determined that 358 genes are over-expressed in the IGF-1/IGF-1R high group (adjusted *p*-value < 0.01). By filtering and analyzing these genes by the Genecodis3 pathway analysis, we found that Focal adhesion is prominent among the 25 enriched KEGG pathways identified (Figure 2A,B), reminiscing the evidence reported in our recent study [22]. Next, querying the METABRIC dataset we found that 1243 genes are over-expressed in the TNBC cohort with high IGF-1/IGF-1R expression levels (adjusted *p*-value < 0.05). Performing the Genecodis3 pathway analysis, Focal adhesion was again assessed as prominent among the 29 enriched KEGG pathways recognized (Figure 2C,D). Taken together, these observations highlight the potential role of the Focal adhesion pathway in the context of the IGF-1/IGF-1R-mediated action in TNBC.

### 3.2. IGF-1/IGF-1R System Triggers FAK Activation

**Figure 3:**
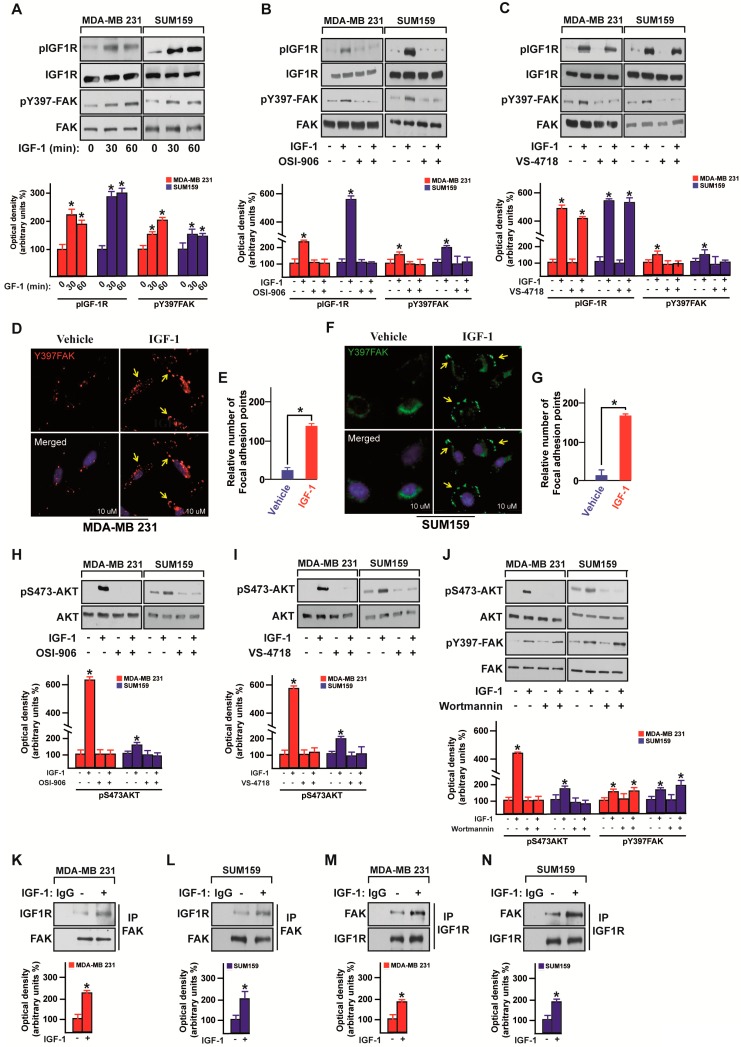
IGF-1 triggers Focal Adhesion Kinase (FAK) activation. (**A**) Immunoblots showing the phosphorylation of both IGF-1R and FAK in MDA-MB 231 and SUM159 TNBC cells upon IGF-1 stimulation (50 ng/mL), as indicated. (**B**) Immunoblots showing the phosphorylation of both IGF-1R and FAK in MDA-MB 231 and SUM159 TNBC cells treated for 30 min with IGF-1 (50 ng/mL) alone or in combination with the IGF-1R inhibitor OSI-906 (1 µM). Underlying panels show densitometric analysis of the immunoblots normalized to the loading controls. (**C**) Immunoblots showing the phosphorylation of both IGF-1R and FAK in MDA-MB 231 and SUM159 TNBC cells treated for 30 min with IGF-1 (50 ng/mL) alone or in combination with the FAK inhibitor VS-4718 (100 nM). Underlying panels show densitometric analysis of the immunoblots normalized to the loading controls. (**D**) Fluorescence images of MDA-MB 231 cells exposed for 30 min to IGF-1 (50 ng/mL). Anti-pY397 FAK staining was detected in red and nuclei were stained with DAPI. Yellow arrows indicate focal adhesions. Scale bars, 10 µm. (**E**) Evaluation of the pY397 FAK fluorescent signal in MDA-MB 231 cells treated with vehicle or IGF-1. (**F**) Fluorescence images of SUM159 cells exposed for 30 min to IGF-1 (50 ng/mL). Anti-pY397 FAK staining was detected in green and nuclei were stained with DAPI. Yellow arrows indicate focal adhesions. Scale bars, 10 µm. (**G**) Evaluation of the pY397 FAK fluorescent signal in SUM159 cells treated with vehicle or IGF-1. (**H**) Immunoblots showing the AKT phosphorylation in MDA-MB 231 and SUM159 TNBC cells treated for 30 min with IGF-1 (50 ng/mL) alone or in combination with the IGF-1R inhibitor OSI-906 (1 µM). Underlying panels show densitometric analysis of the immunoblots normalized to the loading control. (**I**) Immunoblots showing the AKT phosphorylation in MDA-MB 231 and SUM159 TNBC cells treated for 30 min with IGF-1 (50 ng/mL) alone or in combination with the FAK inhibitor VS-4718 (100 nM). Underlying panels show densitometric analysis of the immunoblots normalized to the loading control. (**J**) Immunoblots showing the phosphorylation of both AKT and FAK in MDA-MB 231 and SUM159 TNBC cells treated for 30 min with IGF-1 (50 ng/mL) alone or in combination with the PI3K inhibitor Wortmannin (1 µM). Underlying panels show densitometric analysis of the immunoblots normalized to the loading control. Immunoblots of IGF-1R after immunoprecipitation (IP) of FAK in MDA-MB 231 (**K**) and SUM159 (**L**) cells treated with IGF-1 (50 ng/mL) for 30 min. Western blotting of FAK is also shown. Immunoblots of FAK after immunoprecipitation (IP) of IGF-1R in MDA-MB 231 (**M**) and SUM159 (**N**) cells treated for 30 min with IGF-1 (50 ng/mL). Western blotting of IGF-1R is also shown. Underlying panels show densitometric analysis of the immunoblots normalized to the loading control. Data shown are representative of three independent experiments performed in triplicate. * indicates *p* < 0.05 for cells treated with vehicle versus treatments.

Diverse stimuli such as growth factors and ligands of the G-protein coupled receptors (GPCRs) may induce the activation of FAK, promoting, therefore, cancer cell growth and survival [19]. On the basis of the above-mentioned bioinformatics analysis and considering that the IGF-1R-mediated signaling in cancer may involve FAK [48,49,50], we investigated whether the IGF-1/IGF-1R transduction pathway may lead to FAK phosphorylation in MDA-MB 231 and SUM159 cells that were used as a model system for TNBC. Of note, the IGF-1 induced activation of both IGF-1R and FAK (Figure 3A) was no longer evident in the presence of the IGF-1R inhibitor OSI-906 (Figure 3B), whereas the FAK inhibitor VS-4718 abrogated FAK phosphorylation but not IGF-1R activation (Figure 3C). Nicely corroborating these findings, IGF-1 enhanced the number of focal adhesion points in the MDA-MB 231 (Figure 3D,E) and SUM159 (Figure 3F,G) cells. As the phosphoinositide 3-kinase (PI3K) pathway may trigger the transduction responses mediated by the IGF-1/IGF-1R system in cancer cells [51], we asked whether the PI3K/AKT signaling is involved in the activation of FAK in TNBC cells. First, we observed that both the IGF-1R inhibitor OSI-906 (Figure 3H) and the FAK inhibitor VS-4718 (Figure 3I) prevent the AKT phosphorylation by IGF-1 stimulation in MDA-MB 231 and SUM159 cells. Then, we assessed that the PI3K inhibitor Wortmannin inhibits the AKT activation but not FAK phosphorylation induced by IGF-1 in MDA-MB 231 and SUM159 cells (Figure 3J). Cumulatively, these results suggest that the IGF-1R/FAK/PI3K/AKT transduction cascade may represent a further mechanism through which IGF-1 signals in TNBC. Performing immunoprecipitation assays, we also evidenced that IGF-1 stimulates a direct interaction between IGF-1R and FAK in both MDA-MB 231 and SUM159 cells (Figure 3K–N), further supporting a direct link between the IGF-1/IGF-1R system and FAK action in TNBC.

### 3.3. FAK Is Involved in the IGF-1/IGF-1R-Initiated YAP Activation

**Figure 4:**
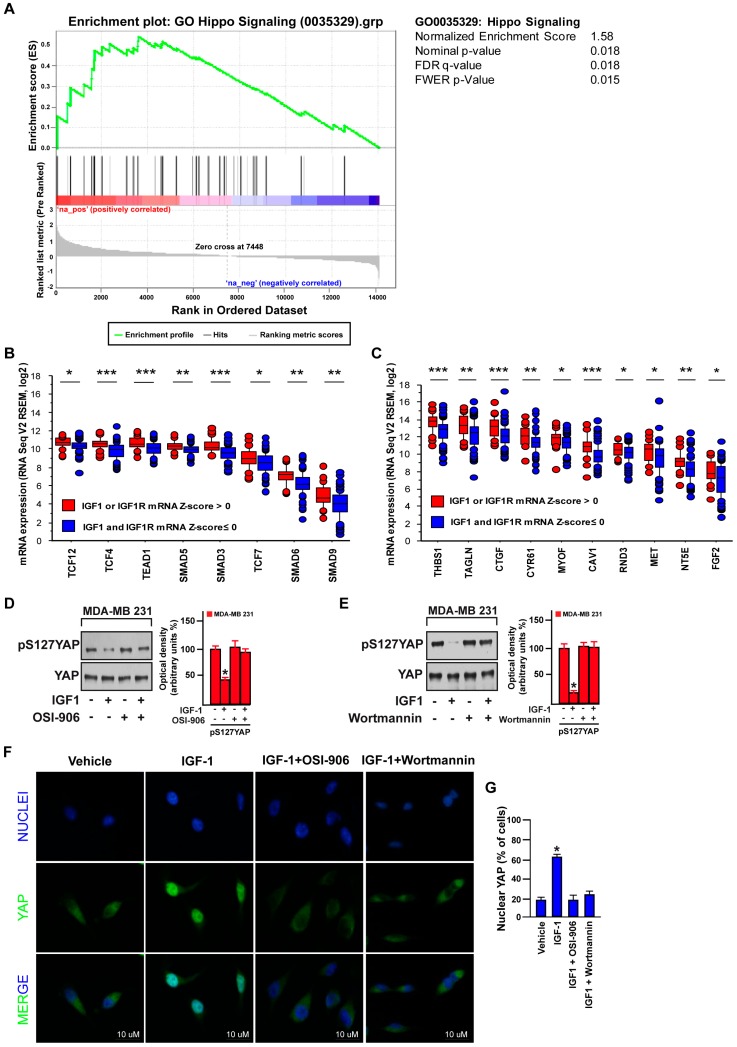
IGF-1 promotes the nuclear accumulation of yes-associated protein/yes-related protein (YAP). (**A**) The Gene Set Enrichment analysis (GSEA) for “Hippo signaling” in Gene Ontology (GO) comparing high and low IGF1/IGF1R groups in TNBC. (**B**) Expression of YAP/TAZ-associated transcriptional factor genes in the TNBC cohort of the TCGA dataset comparing high and low IGF1/IGF1R groups. * *p* < 0.05, ** *p* < 0.01, *** *p* < 0.001. (**C**) Expression of YAP/TAZ target genes in the TNBC cohort of the TCGA dataset comparing high and low IGF1/IGF1R groups. * *p* < 0.05, ** *p* < 0.01, *** *p* < 0.001. Immunoblots showing the phosphorylation of YAP in MDA-MB 231 cells treated for 30 min with IGF-1 (50 ng/mL) alone or in combination with the IGF-1R inhibitor OSI-906 (1 µM). (**D**) or the PI3K inhibitor Wortmannin (1 µM) (**E**). Side panels show densitometric analysis of the immunoblots normalized to the loading control. (**F**) Immunofluorescence staining of YAP (green) and DAPI (blue) in MDA-MB 231 cells treated for 30 min with IGF-1 (50 ng/mL) alone or in combination with the IGF-1R inhibitor OSI-906 (1 µM) or the PI3K inhibitor Wortmannin (1 µM). Scale bars, 10 µm. (**G**) Percentage of MDA-MB 231 cells with nuclear YAP. Data shown are representative of three independent experiments performed in triplicate. Error bars represent mean ± SD. * indicates *p* < 0.05 for cells treated with vehicle versus treatments.

YAP controls the cell proliferation rate and the organ size growth acting as a negative regulator of the evolutionarily conserved Hippo pathway [52]. It has been reported that diverse agents, G protein-coupled receptor (GPCR) ligands [53], like hormones [54,55] and growth factors [56,57], represent potential negative effectors of the Hippo pathway by enhancing the nuclear YAP activity in cancer cells. On the basis of this evidence, we sought to evaluate the potential role of the IGF-1/IGF-1R system in YAP activation in TNBC. By performing a Gene Set Enrichment analysis (GSEA), we ascertained that the “Hippo signaling” signature in Gene Ontology (GO) is significantly enriched TNBC cohorts displaying high IGF-1/IGF-1R expression levels (Figure 4A). Analyzing the TCGA dataset of TNBC, we then found that the expression of YAP/TAZ associated transcriptional factor genes and YAP/TAZ canonical target genes is significantly elevated in patients exhibiting high IGF-1 or IGF-1R (or both) (mRNA Z-score more than 0) respect to patients showing low levels of IGF-1 and IGF-1R (both mRNA Z-scores equal or less than 0) (Figure 4B,C). The activity of YAP is tightly dependent on the phosphorylation status of diverse residues including Serine 127 (S127), which represents one of the most important repressive targets of the Hippo pathway [58]. Taken into account the above-mentioned information, we treated the MDA-MB 231 cells with IGF-1 and revealed its ability to decrease S127 YAP phosphorylation (Figure 4D,E). This effect was no longer evident in the presence of both the IGF-1R inhibitor OSI-906 (Figure 4D) and the PI3K inhibitor Wortmannin (Figure 4E). In accordance with these findings, immunofluorescence studies of nuclear YAP showed that both the IGF-1R inhibitor OSI-906 and the PI3K inhibitor Wortmannin prevent the nuclear accumulation of YAP induced by IGF-1 in MDA-MB 231 cells (Figure 4F,G). Hence, these data provide novel evidence regarding the activation of YAP by the IGF-1/IGF-1R system along with the PI3K/AKT signaling cascade in TNBC cells.

**Figure 5:**
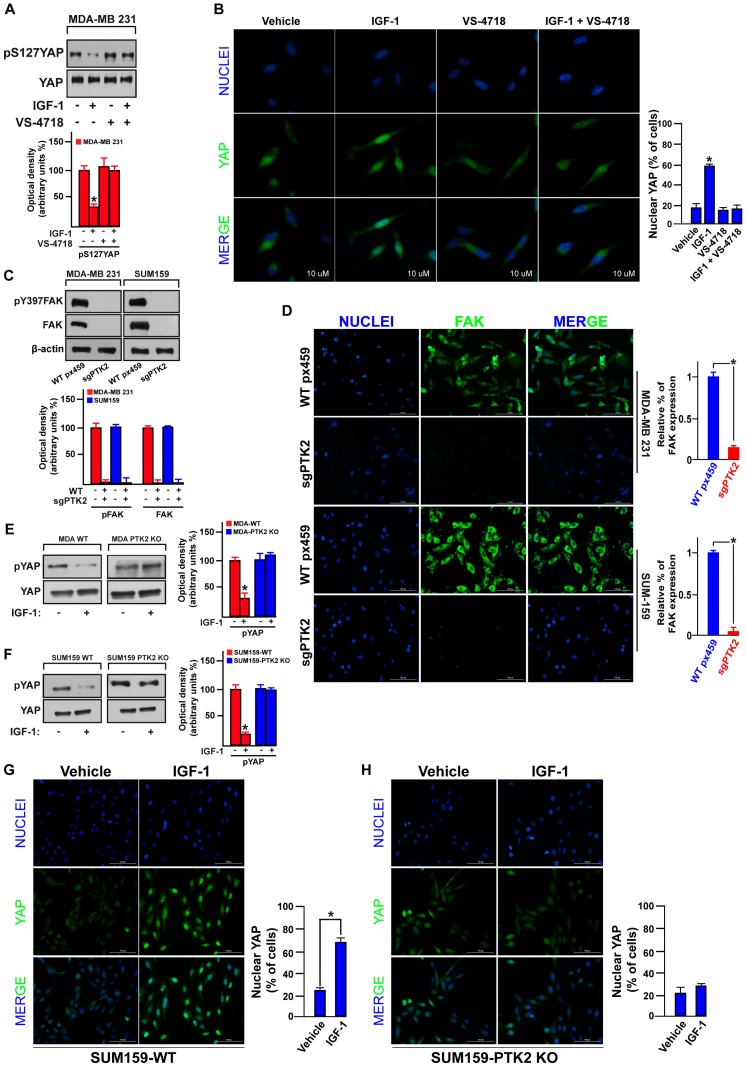
FAK is involved in the IGF-1 induced YAP nuclear translocation. (**A**) Immunoblot showing the phosphorylation of YAP in MDA-MB 231 cells treated for 30 min with IGF-1 (50 ng/mL) alone or in combination with the FAK inhibitor VS-4718 (100 nM). Underlying panels show densitometric analysis of the immunoblots normalized to the loading control. (**B**) Immunofluorescence staining of YAP (green) and DAPI (blue) in MDA-MB 231 cells treated for 30 min with IGF-1 (50 ng/mL) alone or in combination with the FAK inhibitor VS-4718 (100 nM). Scale bars, 10 µm. Percentage of MDA-MB 231 cells with nuclear YAP is also shown. (**C**) Immunoblots showing CRISPR/Cas9-PTK2 knockout in MDA-MB 231 and SUM159 cells. Underlying panels show densitometric analysis of the immunoblots normalized to the loading control. (**D**) Immunofluorescence staining depicting CRISPR/Cas9-PTK2 knockout in MDA-MB 231 and SUM159 cells. Scale bars, 100 µm. FAK fluorescent signal in MDA-MB 231 and SUM159 cells is also shown. Immunoblots showing YAP phosphorylation in MDA-MB 231 WT and PTK2 KO cells (**E**) and in SUM159 WT and PTK2 KO cells (**F**) upon IGF-1 stimulation (50 ng/mL). Side panels show densitometric analysis of the immunoblots normalized to the loading control. Immunofluorescence staining of YAP (green) and DAPI (blue) upon 30 min IGF-1 stimulation (50 ng/mL) in SUM159 WT (**G**) and PTK2-KO cells (**H**). Scale bars, 100 µm. Percentage of SUM159 WT and PTK2-KO cells with nuclear YAP is also shown. Data shown are representative of three independent experiments performed in triplicate. Error bars represent mean ± SD. * indicates *p* < 0.05 for cells treated with vehicle versus treatments.

It has been recently demonstrated that FAK may act as a regulator of the Hippo pathway [31,39,59]. Therefore, we examined the involvement of FAK in the IGF-1/IGF-1R-dependent YAP activation in TNBC. Of note, the blunted S127 YAP phosphorylation (Figure 5A), as well as the YAP nuclear accumulation (Figure 5B) upon IGF-1 exposure, were both rescued using the FAK inhibitor VS-4718 (Figure 5A,B). In order to strengthen these findings, we knocked-out the expression of FAK by CRISPR/Cas9 genome editing technology in both MDA-MB 231 and SUM159 cells (see material and methods section), as proved by western blotting (Figure 5C) and immunofluorescence assays (Figure 5D). This engineered experimental model nicely confirmed that FAK is involved in the IGF-1-mediated YAP activation, as IGF-1 failed to decrease S127 YAP phosphorylation (Figure 5E,F) and YAP nuclear accumulation (Figure 5G,H) in both PTK2-KO MDA-MB 231 and SUM159 cells. Collectively, these data suggest that FAK represents a novel signal transduction mechanism linking the IGF-1/IGF-1R system to YAP activation in TNBC.

### 3.4. FAK Is Involved in the YAP-Dependent Transcriptional Activity Induced by IGF-1/IGF-1R

**Figure 6:**
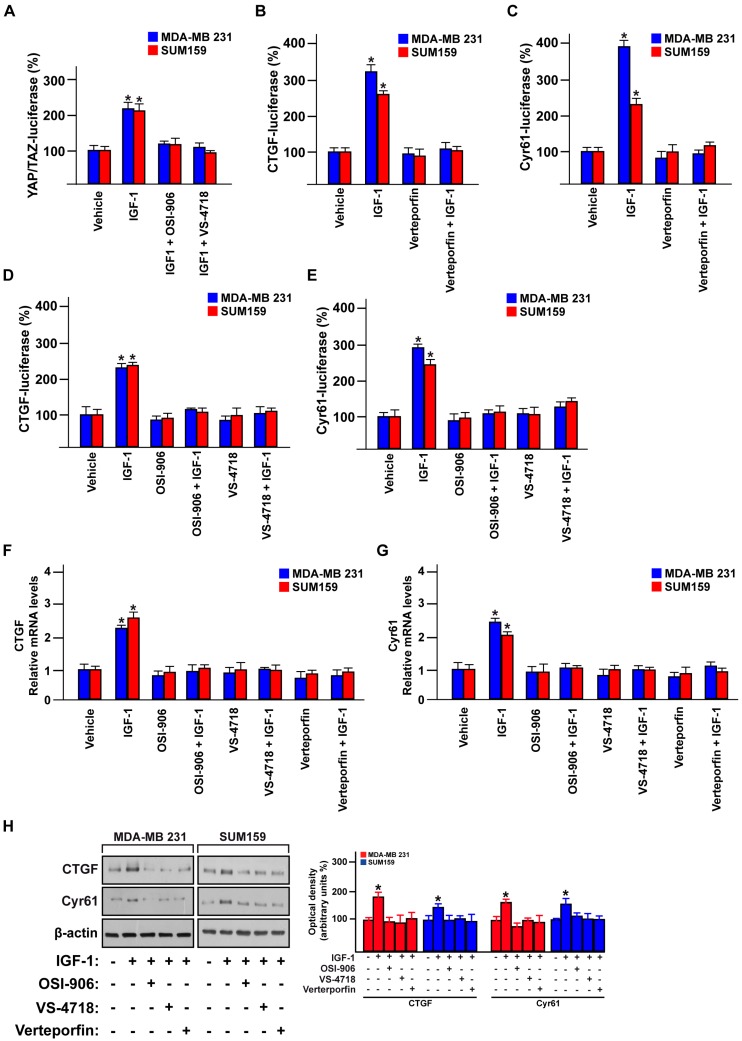
FAK mediates the IGF-1-induced regulation of the YAP target genes CTGF and Cyr61. (**A**) YAP/TAZ luciferase reporter assay in MDA-MB 231 and SUM159 cells treated with IGF-1 (50 ng/mL) alone or in combination with the IGF-1R inhibitor OSI-906 (1 µM) or the FAK inhibitor VS-4718 (100 nM). CTGF (**B**) and Cyr61 (**C**) luciferase reporter assays in MDA-MB 231 and SUM159 cells treated with IGF-1 (50 ng/mL) alone or in combination with the YAP/TEAD complex suppressor Verteporfin (100 nM). CTGF (**D**) and Cyr61 (**E**) luciferase reporter assays in MDA-MB 231 and SUM159 cells treated with IGF-1 (50 ng/mL) alone and in combination with the IGF-1R inhibitor OSI-906 (1 µM) or the FAK inhibitor VS-4718 (100 nM). mRNA expression of CTGF (**F**) and Cyr61 (**G**) in MDA-MB 231 and SUM159 cells treated with IGF-1 (50 ng/mL) alone and in combination with the IGF-1R inhibitor OSI-906 (1 µM), the FAK inhibitor VS-4718 (100 nM) or the YAP/TEAD complex suppressor Verteporfin (100 nM). (**H**) Immunoblots showing the CTGF and Cyr61 protein levels in MDA-MB 231 and SUM159 cells treated with IGF-1 (50 ng/mL) alone and in combination with the IGF-1R inhibitor OSI-906 (1 µM), the FAK inhibitor VS-4718 (100 nM) or the YAP/TEAD complex suppressor Verteporfin (100 nM). Side panels show densitometric analysis of the immunoblots normalized to the β-actin, which was used as loading control. Data shown are representative of three independent experiments performed in triplicate. Error bars represent mean ± SD. * indicates *p* < 0.05 for cells treated with vehicle versus treatments.

YAP is the main Hippo pathway transcriptional co-activator that regulates gene expression changes through the TEAD family members [26,60]. Aiming to further confirm the role exerted by FAK in the IGF-1/IGF-1R-dependent YAP activation in TNBC, we performed YAP/TAZ luciferase reporter assay. Of note, the YAP transcription activity induced by IGF-1 was abolished using either the IGF-1R inhibitor OSI-906 or the FAK inhibitor VS-4718 in both MDA-MB 231 and SUM159 cells (Figure 6A). Next, we sought to investigate whether FAK may affect the expression of the YAP-canonical target genes CTGF and Cyr61 [61]. In this regard, we first assessed whether the suppressor of the YAP/TEAD complex, verteporfin, represses the transactivation of both CTGF and Cyr61 promoter activity triggered by IGF-1 in MDA-MB 231 and SUM159 cells (Figure 6B,C). In addition, the transactivation of the CTGF and Cyr61 promoter sequences by IGF-1 was prevented using the IGF-1R inhibitor OSI-906 and the FAK inhibitor VS-4718 in TNBC cells (Figure 6D,E). In accordance with these findings, the IGF-1R inhibitor OSI-906, the FAK inhibitor VS-4718 and the suppressor of the YAP/TEAD complex verteporfin abolished the expression of CTGF and Cyr61 induced by IGF-1 at both mRNA (Figure 6F,G) and protein levels (Figure 6H). Altogether, these results suggest that IGF-1/IGF-1R may regulate YAP/TEAD target genes in TNBC cells through FAK.

### 3.5. FAK and YAP Contribute to the Growth Responses Induced by IGF-1/IGF-1R

**Figure 7:**
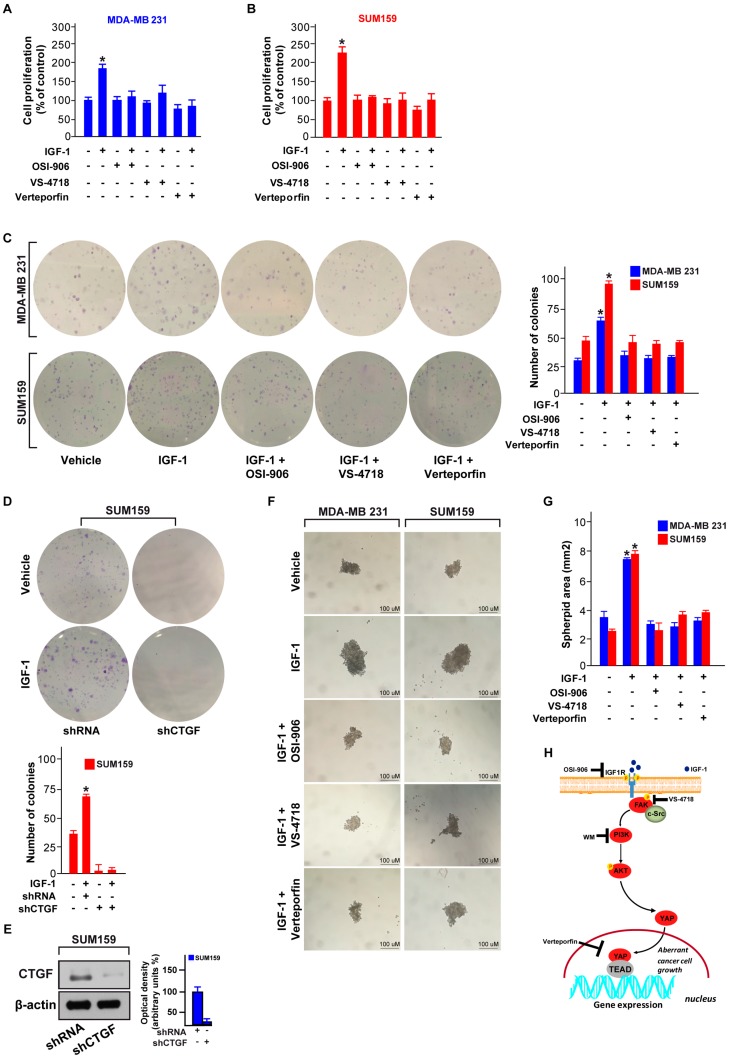
The IGF-1/FAK/YAP signaling pathway triggers the growth of TNBC cells. Proliferation assay of MDA-MB 231 (**A**) and SUM159 (**B**) cells exposed for 4 days to IGF1 (50 ng/mL) alone and in combination with the IGF-1R inhibitor OSI-906 (1 µM), the FAK inhibitor VS-4718 (100 nM) or the YAP/TEAD inhibitor, verteporfin (100 nM). (**C**) Colony formation assay in MDA-MB 231 and SUM159 cells exposed to IGF1 (50 ng/mL) alone and in combination with the IGF-1R inhibitor OSI-906 (1 µM), the FAK inhibitor VS-4718 (100 nM) or the YAP/TEAD inhibitor verteporfin (100 nM). After 10 days of treatment, the plates were stained with crystal violet and colonies were counted, as indicated. (**D**) Colony formation assay in SUM159 cells exposed to IGF-1 (50 ng/mL) and transfected with shRNA or shCTGF. After 10 days of treatment the plates were stained with crystal violet and colonies were counted, as indicated. (**E**) Immunoblot showing the CTGF protein levels in SUM159 cells transfected with control shRNA or shCTGF. Side panels show densitometric analysis of the immunoblot normalized to the β-actin, which was used as loading control. (**F**,**G**) Spheroid formation assay in MDA-MB 231 and SUM159 cells exposed for 18 days to IGF-1 (50 ng/mL) alone and in combination with the IGF-1R inhibitor OSI-906 (1 µM), the FAK inhibitor VS-4718 (100 nM) or the YAP/TEAD complex suppressor verteporfin (100 nM). Scale bars, 100 µm. (**H**) Cartoon depicting the IGF-1/FAK/YAP signaling pathway promoting gene expression changes and the growth of TNBC cells. Data shown are representative of three independent experiments performed in triplicate. Error bars represent mean ± SD. (*) indicates *p* < 0.05 for cells treated with vehicle versus treatments.

Previous studies have reported the growth stimulatory effects elicited by the IGF-1/IGF-1R system toward the proliferation and survival of TNBC cells [17,45]. Hence, we aimed to determine whether the FAK/YAP transduction signaling is involved in growth promotion triggered by IGF-1/IGF-1R system in TNBC cells. In this respect, we assessed that the proliferation (Figure 7A,B), as well as the colony formation ability (Figure 7C) stimulated by IGF-1 in both MDA-MB 231 and SUM159 cells are prevented using the IGF-1R inhibitor OSI-906, the FAK inhibitor VS-4718 and the suppressor of the YAP/TEAD complex, verteporfin. Using these inhibitors alone, the colony formation ability of both MDA-MB 231 and SUM159 cells was similar to that observed in cells treated with vehicle (data not shown). Worthy, the colony formation capability upon IGF-1 exposure was also abrogated by silencing CTGF expression through a specific short-hairpin (shCTGF) in both SUM159 (Figure 7D,E) and MDA-MB 231 (data not shown) cells. Moreover, these inhibitors prevented the spheroid expansion induced by IGF-1 in MDA-MB 231 and SUM159 cells (Figure 7F,G). Using these inhibitors alone, the spheroid expansion of both MDA-MB 231 and SUM159 cells was similar to that observed in cells treated with vehicle (data not shown). Overall, these findings indicate that the IGF-1/IGF-1R-FAK-YAP signal transduction pathway triggers growth stimulatory responses in TNBC cells, as depicted in Figure 7H.

## 4. Discussion

The generation of bioinformatics pipelines led to the recognition of suitable connections among signaling networks and gene expression signatures toward the assessment of molecular vulnerabilities useful as novel cancer drug targets [34]. Within the multifaceted human diseases, the breast cancer subtype named TNBC may represent a good testbed to apply bioinformatics analyses in order to discover new molecular mechanisms and therapeutics in this aggressive human malignancy [62]. On the basis of these challenges, we began the current study processing the data available on the vast public databases Cancer Genome Atlas (TCGA) and Molecular Taxonomy of Breast Cancer International Consortium (METABRIC). Our analysis revealed that a high expression of IGF-1 or IGF-1R (or both) does not affect the OS or DFS rates in patients with ER/PR-positive and HER2-negative breast cancer subtype, but it associates with a reduced DFS rate in TNBC patients. By the thorough dissection of the transduction networks linked to high IGF-1/IGF-1R expression in the TNBC cohort of these datasets, we identified the focal adhesion as a prominent and enriched pathway, laying the rationale to investigate the potential of FAK in the TNBC progression. Worthy, we ascertained that the IGF-1/IGF-1R system triggers FAK activation, increases FAs and engages thereafter PI3K/AKT signaling towards YAP nuclear accumulation. Further corroborating these findings, a bioinformatic analysis evidenced that the Hippo cascade and its related target genes represent key transduction pathways in the high IGF-1 or IGF-1R TNBC group. Using different pharmacological approaches and genome editing technology, we also demonstrated that FAK inhibition prevents YAP-dependent transcriptional activity and gene expression upon IGF-1 stimulation in TNBC cells. Our studies also revealed the growth suppressive activity of interfering with the IGF-1/IGF-1R/FAK/YAP signal transduction network in TNBC, which therefore may provide novel therapeutic approaches for the treatment of TNBC.

TNBC still represents a breast cancer subtype characterized by a worse prognosis and high risk of relapse due to a limited spectrum of therapeutic options [63]. Diverse frequency mutation rates (i.e., *TP53* and *PIK3CA*), as well as gene and/or mRNA over-expression, may contribute to the TNBC development and drug-resistance [32]. In this regard, the portrait of genetic alterations in breast cancer support a dysfunction of the IGF-1/IGF-1R system leading to the development of various breast malignancies, including TNBC [32,43]. In particular, it has been shown that approximately 30–40% of TNBCs harbors amplification of the IGF-1R gene, which was linked to a short survival rate of these patients [16,64]. Moreover, the signaling capacity and proliferative responses mediated by IGF-1, as well as overall IGF-1/IGF-1R activity, were found to be elevated in certain TNBCs [8,45]. Collectively, these findings highlight the contribution of the IGF-1/IGF-1R system in the progression of TNBC in agreement with our current data.

Previous studies have demonstrated that FAK may be involved in the development of various types of tumors, including breast cancer [65]. Of note, FAK over-expression has been tightly correlated with the malignant grade [66] as well as the poor clinical outcome in TNBC patients [22]. Moreover, FAK activation was involved in the peculiar features of TNBC [65], whereas the inhibition of FAK signaling prevented the metastatic potential of TNBC cells [22,23] and enhanced the sensitization to anti-cancer treatments [67]. However, the precise mechanism by which FAK acts in TNBC are still poorly understood. In addition to its canonical functions, FAK contributes to diverse growth factors-mediated signaling toward cancer cell proliferation and dissemination [68,69]. For instance, the IGF-1/IGF-1R system may associate with FAK toward the activation of downstream pathways and cell survival, proliferative and migratory functions in different tumors, including TNBC [50,70,71]. In line with these findings, our study identified focal adhesion as a prominent pathway among the high IGF-1 or IGF1R TNBC cohort and ascertained a direct cooperation between the IGF-1/IGF-1R system and FAK in TNBC cells.

The Hippo/YAP signaling exerts important regulatory effects in diverse pathophysiological conditions, including breast cancer [28,72]. For instance, in non-transformed MCF-10A breast cancer cells, the activation of YAP determines an EMT-like phenotype and promotes cell proliferation without growth factors stimulation [73]. Moreover, YAP over-expression was involved in the onset and metastasis of breast cancer [74], whereas YAP inhibition diminished both the lung-metastasis recurrence in an engineered mouse model of breast cancer and rescued the radio-resistance of TNBC cells [30,75]. In the framework of the abovementioned observations, it is worth mentioning that the IGF-1/IGF-1R system behaved like a modulator of the hypoxia-activated YAP signaling in hepatocellular carcinoma [57]. Besides, it should be noted that FAK contributed to both the YAP-dependent cell proliferation/differentiation during mouse development [76] and the mechanical YAP activation [77]. Recently, our synthetic platform of lethal gene interactions also highlighted the role of FAK in the growth of YAP-dependent uveal melanoma [39]. In accordance with these findings, in the current study, we ascertained that the Hippo pathway and its related transcription factors are significantly enriched in the high IGF-1 or IGF-1R TNBC group. In addition, we showed that in TNBC cells the activation of the IGF-1/IGF-1R and FAK signaling triggers the nuclear accumulation of YAP and its transcriptional function. Biologically, the inhibition of IGF-1/IGF-1R, FAK or YAP abolished the growth of TNBC cells, as evaluated in multiple experimental systems. It is worth mentioning that the IGF-1R, FAK and YAP/TAZ inhibitors used in the present study, enhanced the efficacy of diverse chemotherapeutics in different tumor contexts [78,79,80] further corroborating their usefulness as antitumor agents. In the framework of these observations, it should be noted that IGF-1 can also engage IR in mediating stimulatory effects in TNBC cells, albeit to a reduced extent due to the lower expression of IR with respect to IGF-1R [45,81]. In order to overcome this promiscuous response to IGF-1, the dual IGF-IR/IR inhibitor OSI-906 was developed to halt the transduction signaling mediated by both receptors [81]. Hence, future studies are warranted to rule out the peculiar contribution of IR in the action of IGF-1 in TNBC cells, as observed in the present study.

Overall, our results suggest that the IGF-1/IGF-1R-FAK-YAP transduction pathway may trigger growth stimulation of TNBC cells. However, further investigations are needed to better address the functional interaction and the biological outcomes orchestrated by these emerging players, particularly considering that the TNBC is a multifaceted malignancy characterized by different subtypes and phenotypes [82].

## Abbreviations

TNBC	triple-negative breast cancer
TCGA	The Cancer Genome Atlas
METABRIC	Molecular Taxonomy of Breast Cancer International Consortium
OS	overall survival
DFS	disease free survival
GSEA	gene set enrichment analysis
IGF-1	insulin-like growth factor-1
IGF-1R	insulin-like growth factor-1 receptor
IR	insulin receptor
PI3K	phosphatidylinositol 3-kinase
AKT	protein kinase B
FAK	focal adhesion kinase
YAP	yes-associated protein
TEAD	TEA domain family member
CRISPR	clustered regularly interspaced short palindromic repeats
sgRNA	single guide RNA
KO	knock out
WT	wild type
shRNA	short hairpin RNA
CTGF	connective tissue growth factor
Cyr61	cellular communication network factor 1/cysteine-rich angiogenic inducer 61
