# Correction: Rigiracciolo, D.C., et al., IGF-1/IGF-1R/FAK/YAP Transduction Signaling Prompts Growth Effects in Triple-Negative Breast Cancer (TNBC) Cells. *Cells* 2020, 9, 1010

**DOI:** 10.3390/cells9122619

**Published:** 2020-12-06

**Authors:** Damiano Cosimo Rigiracciolo, Nijiro Nohata, Rosamaria Lappano, Francesca Cirillo, Marianna Talia, Domenica Scordamaglia, J. Silvio Gutkind, Marcello Maggiolini

**Affiliations:** 1 Department of Pharmacy, Health and Nutritional Sciences, University of Calabria, 87036 Rende, Italy; damianocosimo.rigiracciolo@unical.it (D.C.R.); rosamaria.lappano@unical.it (R.L.); francesca.cirillo@unical.it (F.C.); marianna.talia@unical.it (M.T.); scordamagliadomenica1@gmail.com (D.S.); 2 MSD K.K., Tokyo 102-8667, Japan; nijiro.nohata@merck.com; 3 Department of Physics, University of Calabria, 87036 Rende, Italy; 4 Department of Pharmacology, Moores Cancer Center, University of California, San Diego, La Jolla, CA 92093, USA

The authors wish to make the following changes to their paper [1]. Due to the authors having made an error, the last panel of the MDA-MB 231 group of Figure 7C needs to be corrected. Figure 7 should be changed from:

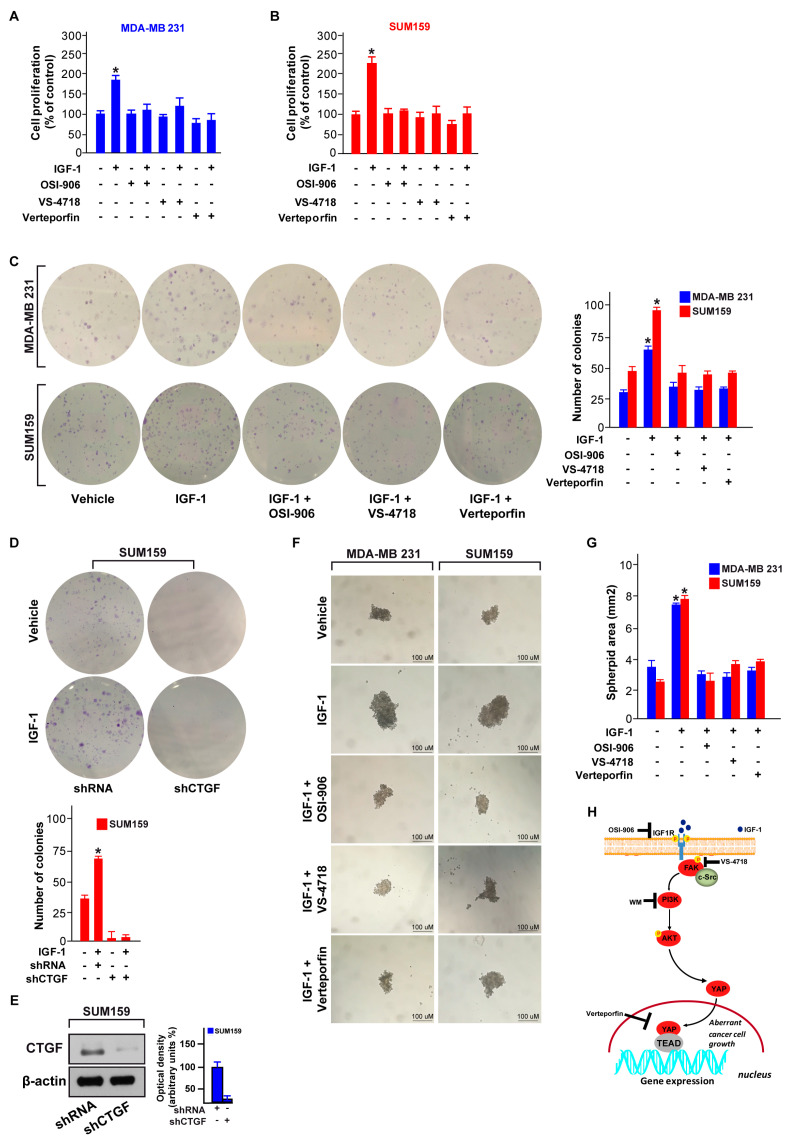



to:

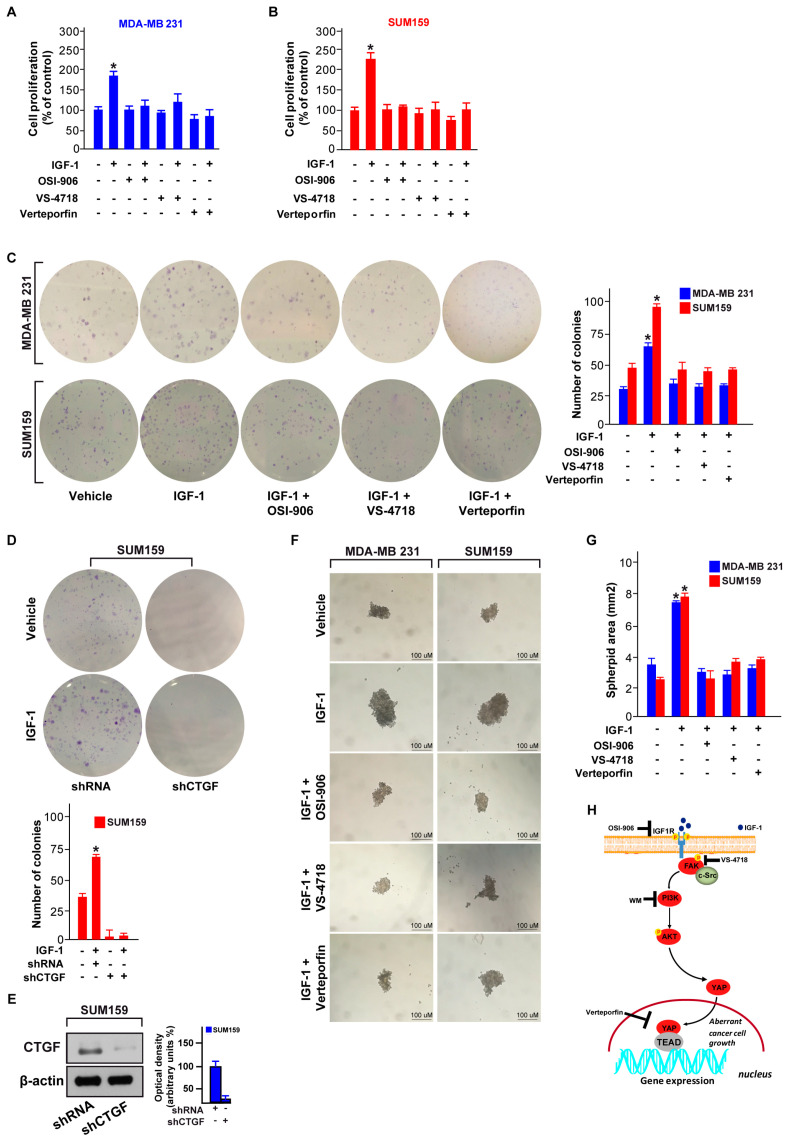



The authors apologize for any inconvenience caused and state that the scientific conclusions are unaffected. The original article has been updated. All changes have been reviewed and approved by the Academic Editors of *Cells*.
